# Double-Access Strategy for Chronic Portal Vein Thrombosis: Salvage Recanalization via Transsplenic and Transhepatic Routes

**DOI:** 10.7759/cureus.92457

**Published:** 2025-09-16

**Authors:** Jose D Cardona Ortegón, Aura Ramirez, Laura Manuela Olarte Bermúdez, David Torres, Oscar Rivero

**Affiliations:** 1 Radiology, University Hospital Fundación Santa Fé de Bogotá, Bogotá, COL

**Keywords:** cavernous degeneration, gastrointestinal bleeding, liver transplant, portal hypertension, portal thrombosis, radiological intervention

## Abstract

Chronic portal vein thrombosis (PVT) with cavernous transformation is a rare but serious complication after liver transplantation, often associated with portal hypertension and gastrointestinal (GI) bleeding. We report the case of a 47-year-old female liver transplant (LT) recipient with recurrent bleeding and imaging findings of chronic PVT. Despite extensive evaluation, no active bleeding source was identified. Given persistent symptoms and portal hypertensive changes, portal vein recanalization (PVR) was attempted. A combined transhepatic and transsplenic approach enabled through-and-through access across the occlusion. Sequential balloon angioplasty and deployment of a covered stent with overlapping bare-metal stents restored portal venous flow and reduced collateral circulation. Technical success was achieved, with no major complications apart from a self-limited perisplenic hematoma managed with embolization. This case demonstrates the feasibility and effectiveness of a dual-access endovascular strategy for complex chronic PVT with cavernous transformation in post-transplant patients.

## Introduction

Portal vein thrombosis (PVT) after liver transplantation is an uncommon but clinically significant complication, with an incidence of 1-3% in adults and up to 5.7% in pediatric recipients [1-3]. This condition can lead to serious manifestations of portal hypertension, including ascites, variceal formation, gastrointestinal (GI) bleeding, graft dysfunction, and even graft failure. Early identification and effective treatment are critical to reduce morbidity and mortality. Management of PVT in the post-transplant setting can be challenging, particularly in chronic cases with cavernous transformation that distorts normal portal venous anatomy and impedes standard recanalization techniques. Traditional endovascular approaches include transhepatic, transjugular, or transsplenic routes individually. However, complex occlusions may require a combined “double-access” strategy, utilizing both transhepatic and transsplenic approaches to establish through-and-through access, facilitate guidewire passage across the occlusion, and enable effective angioplasty and stent deployment.

In cases of chronic PVT after liver transplantation, associated findings such as splenomegaly, ascites, and variceal dilation of the portal venous system often demonstrate significant structural changes. The native portal lumen may be completely obliterated and replaced by multiple periportal collateral veins, a process known as cavernous transformation [4]. Commonly dilated tributaries include the cystic and pericholecystic veins. This altered anatomy eliminates the normal proximal-to-distal continuity of the portal vein, preventing direct guidewire passage. While single vascular access may suffice in patients with preserved venous channels, it frequently fails when the vein is chronically occluded or replaced by cavernous collaterals. In such scenarios, particularly in patients presenting with complications of portal hypertension such as ascites or GI bleeding, portal vein recanalization (PVR) becomes a therapeutic option. Several endovascular techniques have been described; however, the combined use of transhepatic and transsplenic access can create a novel through-and-through pathway that facilitates guidewire crossing, angioplasty, and stent placement. Published reports have demonstrated the feasibility and safety of this double-access strategy, establishing it as an important alternative when conventional single-access approaches are unsuccessful [3,5].

Traditional endovascular approaches include transhepatic, transjugular, or transsplenic routes individually. In the broader management of post-transplant PVT, initial strategies often include anticoagulation, followed by shunt-based procedures such as transjugular intrahepatic portosystemic shunt (TIPS) or PVR with TIPS (PVR-TIPS). Endovascular recanalization techniques are typically considered when these measures are insufficient or anatomically unfeasible. We present the case of a post-transplant patient with chronic PVT successfully managed with combined transhepatic and transsplenic PVR and stent placement. Our objective is to describe the technical steps, immediate outcomes, and periprocedural complications of a dual-access salvage recanalization in a post-liver transplant (LT) patient.

## Case presentation

A 47-year-old female patient with a history of liver transplantation in 2023 due to cirrhosis secondary to bile duct injury presented with recurrent episodes of upper GI bleeding. Basic laboratory workup revealed stable hemoglobin levels with no evidence of coagulopathy, and Doppler ultrasound confirmed absent flow in the main portal vein, raising suspicion for chronic thrombosis. She had known post-transplant chronic PVT and cavernous transformation, resulting in prehepatic portal hypertension and esophageal varices, which had required multiple endoscopic interventions, including band ligation. She was admitted to the emergency department with a new episode of upper GI bleeding. On admission, she was hemodynamically unstable, requiring intensive care unit (ICU) management. Despite multiple endoscopic and capsule evaluations, no active bleeding source was identified, raising concern for portal hypertensive enteropathy as the underlying cause of recurrent unexplained bleeding.

During hospitalization, she experienced a second episode of upper GI bleeding. However, capsule endoscopy showed non-active bleeding of unclear origin; upper endoscopy ruled out esophageal or gastric variceal sources. At this point, the clinical problem statement was summarized as recurrent unexplained GI bleeding in an LT recipient with chronic PVT and cavernous transformation.

**Figure 1:**
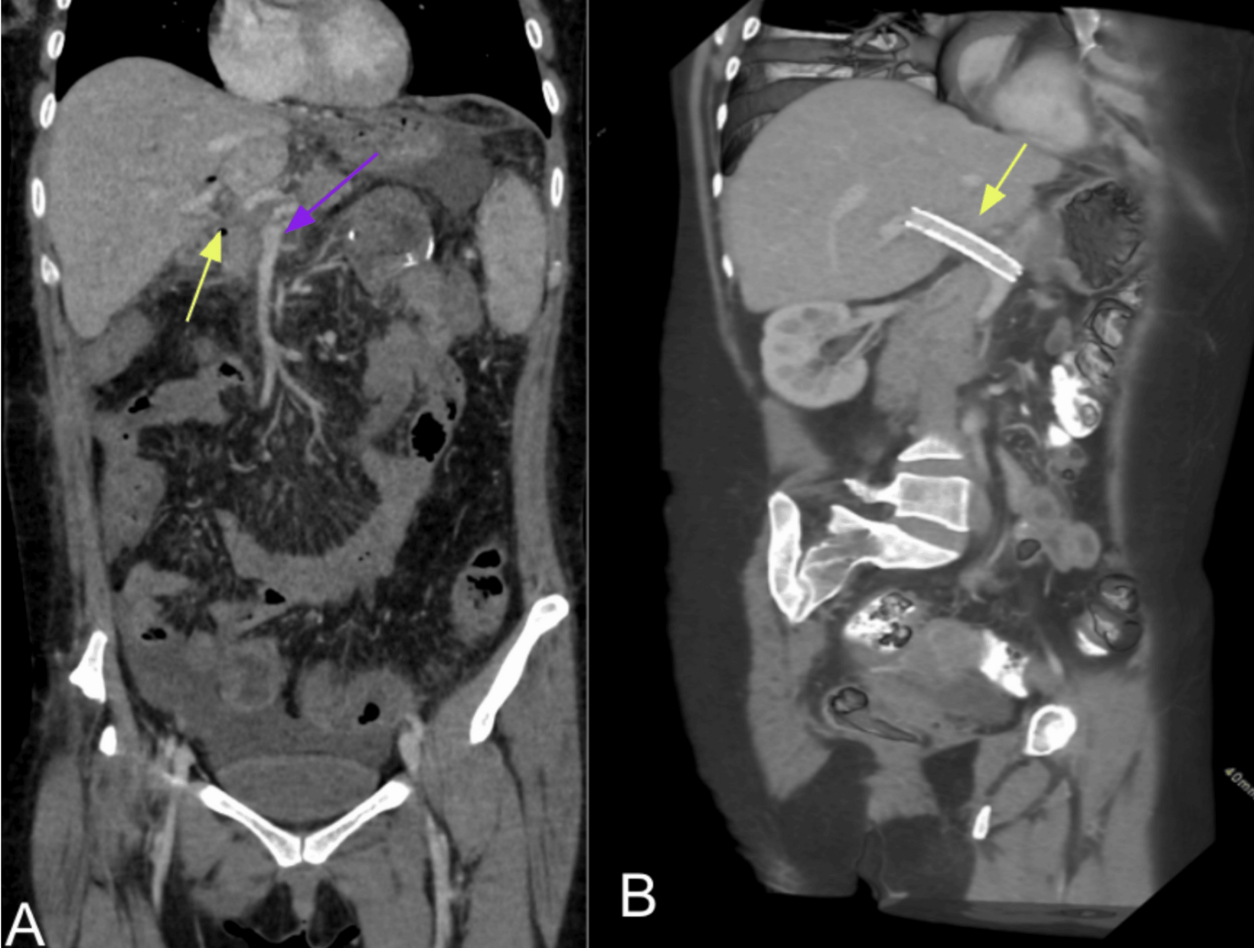
Contrast-enhanced abdominal CT angiography (A) Coronal reconstruction shows diffuse colitis involving the ascending, transverse, descending, and sigmoid colon, associated with pericolic fat stranding. The liver graft demonstrates atrophy of the left hepatic lobe (purple arrow) with compensatory hypertrophy of the right lobe. Cavernous transformation of the portal vein is identified (yellow arrow), secondary to chronic thrombosis, with no evidence of main portal vein recanalization. Additional signs of portal hypertension include perisplenic collateral vessels, splenomegaly, perigastric and mesenteric varices, and ascites in the perihepatic, perisplenic, paracolic, and pelvic spaces. (B) Sagittal reconstruction depicts a metallic stent at the hepatic hilum (yellow arrow), corresponding to a previously placed transjugular intrahepatic portosystemic shunt (TIPS), as part of the therapeutic strategy for portal vein thrombosis

Despite extensive evaluation, including laboratory tests, upper endoscopy, and colonoscopy, the source of bleeding remained unidentified. A contrast-enhanced abdominal computed tomography (CT) scan (Figure 1A) confirmed chronic, non-recanalized PVT with cavernous transformation but no evidence of active bleeding. Hepatology consultation confirmed preserved liver graft function, with no signs of graft dysfunction. Given the persistent bleeding and the inability to identify the source, PVR was considered in an attempt to reduce the effects of portal hypertensive enteropathy.

**Figure 2:**
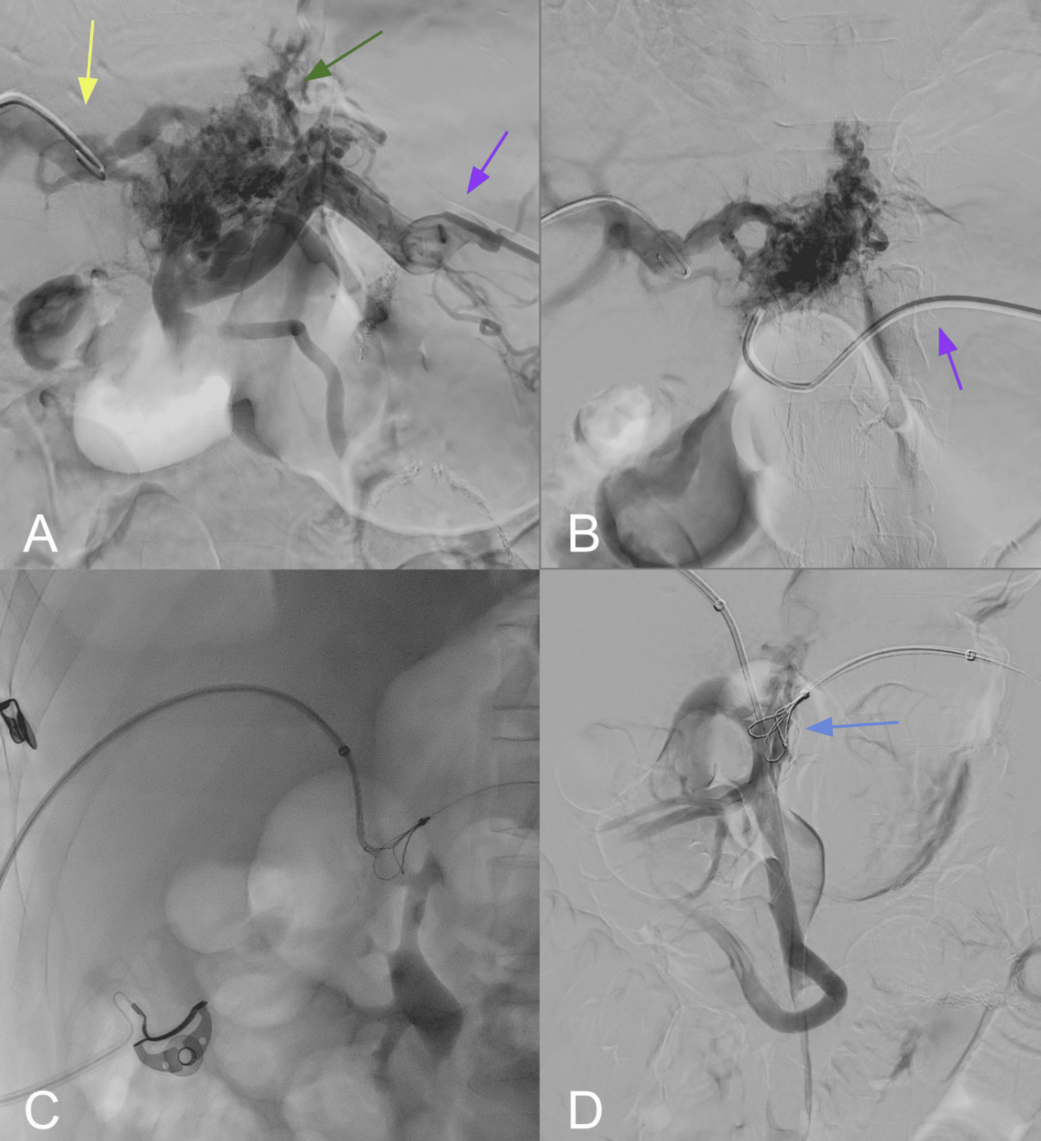
Splenoportography, transhepatic access, and portal vein recanalization (A) Contrast injection through the splenic vein (purple arrow) and the left portal vein (yellow arrow) reveals a complex network of varices arising from the left gastric vein (green arrow), consistent with cavernomatous transformation. (B) Selective catheterization of the left gastric vein via the splenic vein confirms its communication with the left portal vein. (C) Through a right transhepatic approach, a vascular sheath is positioned, allowing advancement of guidewires in preparation for portal vein stenting. (D) Successful cannulation of the superior mesenteric vein is achieved (blue arrow), facilitating the establishment of through-and-through access for endovascular reconstruction

PVR was attempted via combined transhepatic and transsplenic access. Initial angiography demonstrated complete occlusion of the main portal vein with prominent, tortuous periportal collateral vessels, consistent with cavernomatous transformation (Figure 2). Endovascular reconstruction was achieved by advancing a 0.035" guidewire through the transhepatic sheath, which was snared using a loop catheter introduced via transsplenic access at the splenoportal confluence (Figure 2A). After establishing a through-and-through access between the main portal vein and the splenoportal confluence, sequential tract dilations were performed using a 7 mm angioplasty balloon (Figure 2B). A covered stent was deployed, followed by two overlapping bare-metal stents (each 9 mm in diameter) proximally and distally, reestablishing portal vein patency while preserving inflow and outflow from the splenoportal axis (Figure 2C).

**Figure 3:**
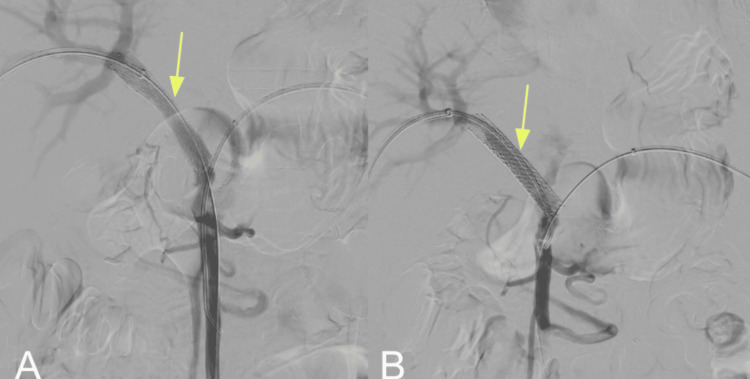
Splenoportography following transhepatic and transsplenic portal vein recanalization (A) Non-expanding perisplenic hematoma measuring approximately 90 × 40 mm was identified as an immediate post-procedural complication. Management included percutaneous embolization of the transsplenic access tract and prophylactic arterial embolization of distal branches of the third and lower splenic segments to prevent further hemorrhagic complications

Final angiographic control demonstrated technical success, with restoration of portal venous flow through the stent, absence of residual thrombosis, and a significant reduction in periportal collaterals, consistent with effective PVR (Figure 3).

## Discussion

Interventional radiology techniques, such as percutaneous transhepatic angioplasty and stent placement, have emerged as effective therapeutic options for the management of PVT. In the present case, a combined transhepatic and transsplenic approach was utilized to achieve successful PVR. Using 0.035" and 0.018" angiographic guidewires and a 7 mm angioplasty balloon, the transvenous tract was dilated, followed by the deployment of a covered stent. This strategy allowed for restoration of portal venous flow while preserving patency of the afferent and efferent vessels of the splenoportal axis. Such combined access techniques have been reported as effective alternatives for restoring portal flow, particularly in cases where the transhepatic route alone proves technically challenging or insufficient [2]. Angioplasty and stenting are cornerstone interventions for the treatment of PVT, particularly in post-transplant patients, to prevent severe portal hypertension and its associated complications. Growing evidence supports the efficacy of combined approaches, such as transhepatic and transsplenic access, in overcoming the technical challenges posed by complete occlusion or cavernous transformation of the portal vein. These techniques have been associated with improved clinical outcomes. For instance, Zeydanli et al. reported a 100% technical success rate for endovascular treatment of portal vein stenosis following liver transplantation [3-6].

Clinical and imaging follow-up after PVR is essential to detect early restenosis and to monitor the control of portal hypertension. Recommended measures include regular clinical evaluation for recurrent bleeding, ascites, or encephalopathy, laboratory assessment of liver function, and endoscopic surveillance when high-risk varices are present. Imaging protocols commonly involve Doppler ultrasound within 24 hours, at one week, and at one month, followed by 6-12-month intervals. Computed tomography angiography (CTA) is typically reserved for patients with abnormal Doppler findings or recurrent symptoms. This strategy has been associated with long-term stent patency rates and regression of varices. Other protocols recommend Doppler evaluation on day 1, and at one, three, and six months, followed by semiannual assessments, escalating to CTA, or, when indicated, direct portography in cases of equivocal Doppler findings, with durable outcomes reported at extended follow-up. Antithrombotic strategies remain heterogeneous. While some groups advocate therapeutic low-molecular-weight heparin for six months with discontinuation if no additional prothrombotic risk factors are present, others prefer heparin bridging followed by warfarin for at least six months, maintaining an international normalized ratio (INR) of 2.0-2.5. In addition, follow-up strategies must be tailored to clinical and procedural circumstances. In patients with transsplenic access or minor periprocedural complications (such as perisplenic hematoma treated with embolization), early imaging during the first 4-6 weeks is particularly emphasized to ensure sustained hepatopetal flow and regression of collateral circulation [7,8].

Successful PVR using a combined transsplenic and transhepatic approach with stent placement underscores the importance of meticulous technical execution and coordinated multidisciplinary management in the treatment of complex PVT following liver transplantation. Imaging modalities are fundamental throughout both the initial diagnostic workup and post-procedural follow-up. Techniques such as Doppler ultrasound, CT, and magnetic resonance imaging (MRI) are essential for assessing the extent of thrombosis and identifying collateral vessel formation. Among imaging modalities, contrast-enhanced MRI is particularly valuable in detecting cavernous transformation and guiding therapeutic decision-making.

## Conclusions

PVT after liver transplantation continues to be a complex and challenging condition to manage. In selected patients, interventional procedures that combine different vascular access routes with advanced recanalization techniques can provide an alternative when conventional approaches are insufficient. The present case shows that using dual-access and complex endovascular strategies may achieve technical success with favorable short-term results. Although this strategy demonstrates potential as an alternative when single-access approaches fail, further studies and long-term follow-up are needed to establish its definitive effectiveness. Moreover, the case underscores the relevance of multidisciplinary management and the essential role of modern imaging in supporting therapeutic decisions and improving patient outcomes.
